# The detection of delirium in admitted oncology patients: a scoping review

**DOI:** 10.1007/s41999-021-00586-1

**Published:** 2022-01-15

**Authors:** Megan B. Sands, Ian Wee, Meera Agar, Janette L. Vardy

**Affiliations:** 1 University of New South Wales Prince of Wales Clinical School, Sydney, Australia; 2 Singapore University Medical School, Singapore, Singapore, Singapore; 3 University of Technology Sydney, Sydney, NSW Australia; 4 Concord Cancer Centre, Concord Repatriation General Hospital, Sydney, NSW Australia; 5 Sydney Medical School, University of Sydney, Sydney, NSW Australia

**Keywords:** *Delirium*, Oncology, Cancer, Inpatient, Detection, Screening

## Abstract

**Purpose:**

Delirium leads to poor outcomes for patients and careers and has negative impacts on staff and service provision. Cancer rates in elderly populations are increasing and frequently, cancer diagnoses are a co-morbidity in the context of frailty. Data relating to the epidemiology of delirium in hospitalised cancer patients are limited. With the overarching purpose of improving delirium detection and reducing the morbidity and mortality of delirium in cancer patients, we reviewed the epidemiological data and approach to delirium detection in hospitalised, adult oncology patients.

**Methods:**

MEDLINE, EMBASE, CINAHL, PsycINFO, and SCOPUS databases were searched from January 1996 to August 2017. Key concepts were delirium, cancer, inpatient oncology and delirium screening/detection.

**Results:**

Of 896 unique studies identified; 91 met full-text review criteria. Of 12 eligible studies, four applied recommended case ascertainment methods to all patients, three used delirium screening tools alone or with case ascertainment tools sub-optimally applied, four used tools not recommended for delirium screening or case ascertainment, one used the Confusion Assessment Method with insufficient information to determine if it met case ascertainment status. Two studies presented delirium incidence rates: 7.8%, and 17% respectively. Prevalence rates ranged from 18–33% for general medical or oncology wards; 42–58% for Acute Palliative Care Units (APCU); and for older cancer patients: 22% and 57%. Three studies reported reversibility; 26% and 49% respectively (APCUs) and 30% (older patients with cancer). Six studies had a low risk of bias according to QUADAS-2 criteria; all studies in the APCU setting were rated at higher risk of bias. Tool selection, study flow and recruitment bias reduced study quality.

**Conclusion:**

The knowledge base for improved interventions and clinical care for adults with cancer and delirium is limited by the low number of studies. A clear distinction between screening tools and diagnostic tools is required to provide an improved understanding of the rates of delirium and its reversibility in this population.

**Supplementary Information:**

The online version contains supplementary material available at 10.1007/s41999-021-00586-1.

## Key summary points

**Aim:**

To understand the validation of delirium detection tools in medical oncology, as well as identify data on incidence, prevalence and reversibility in this setting.

**Findings:**

Of twelve studies, only four used case ascertainment methods following published recommendations, six studies had a low risk of bias.

**Message:**

In delirium tool validation studies in the oncology setting, choice of appropriate gold standard for case ascertainment is a critical factor. New tools and new validations are not recommended, rather the critical application of existing tools depending on appropriate validation and clinical practicality for the setting.

**Supplementary Information:**

The online version contains supplementary material available at 10.1007/s41999-021-00586-1.

## Background and aim

Delirium is a neurocognitive syndrome characterised by an altered level of arousal, altered awareness and cognition, and a reduced ability to direct, focus, sustain, and shift attention [1, 2]. Delirium is associated with increased morbidity and mortality, longer length of stay [3, 4], and marked distress for cancer patients, their families and staff [5, 6]. Delirium is common in hospitalised patients [1, 2], and outcomes can be improved via prevention [7, 8] and effective management [9]. The use of validated assessments improves detection and provides earlier identification of patients with delirium [10].

Under-diagnosis of delirium is an important issue in clinical settings [11]; outcomes are worse if the diagnosis of delirium is delayed or missed entirely [12]. Studies of general hospital patients indicate that pain, younger age, correct orientation in person, place and time, and previous psychiatric diagnosis, especially bipolar disorder or psychosis, are important risk factors for the diagnosis of delirium being missed [13]. One study has shown increasing age, poor performance on cognitive testing and lower serum albumin to be associated with a higher risk of delirium in the hospitalised cancer patients, however, less is known about factors which increases the misdiagnosis of delirium in cancer populations or whether there are specific clinical factors which can be used to mitigate risk [3].

The majority of epidemiological studies in delirium have targeted people over 65 years of age [14]. Although guidelines for the management of delirium in cancer settings exist [15], fewer studies have primarily focussed on adults (defined as 18yrs or older) in an acute hospital, oncology, inpatient setting [16–18]. More commonly studies including cancer patients have been in “stand-alone” palliative care units [19, 20], or subsets of cancer inpatient cohorts on the basis of palliative care [21–23] or psychiatry consultation/liaison services in acute hospitals [11]. A recent review of delirium in the palliative care setting yielded a point prevalence estimate of 35% [95% confidence interval (CI) = 0.29–0.40] at inpatient admission. [20] Studies indicate that in the palliative care cancer setting at least, whilst the prevalence of delirium is high, it remains reversible in approximately half of cases [24]. These data also lend support to the case for improved detection. Of interest, reversibility in the palliative care setting although not a universal possibility, has been associated with factors such as delirium aetiology specifically opioid, or other psychoactive medication, or dehydration, and where there is a less severe cognitive disturbance or absence of organ failure [9, 25].

We chose a scoping review methodology because initial searches yielded few returns in the target setting. We also chose to take a broad approach to clarify key concepts in delirium detection in cancer settings and identify key concepts and gaps in the evidence base [26]. Our review explores the literature in relation to delirium detection and missed delirium in the inpatient oncology setting, and clinical factors associated with misdiagnosis. The aim of this scoping review is to synthesise knowledge and identify gaps relating to detection tool selection, incidence, prevalence and reversibility of delirium in hospitalised, adult patients with cancer.

## Patients and methods

The target population was admitted, adult, oncology patients in an acute-hospital or comprehensive cancer centre. The research questions were:

1. Which instruments are most commonly used to detect delirium?
2. Which reference standards have been used to measure rates of delirium and compare the performance of delirium screening instruments?
3. What is the incidence and prevalence of delirium in the target setting? and
4. What is the rate of reversibility of delirium in the target setting?

Our search strategy centred on four key domains; delirium, cancer, inpatient oncology, and delirium detection. Full inclusion criteria were: original study, English language, for inclusion the focus of the study must be syndromic delirium e.g. not: confusion, cognitive impairment, acute brain syndrome. The target population is patients with cancer and the setting is adult inpatient oncology, studies not relevant to this population were excluded. Specifically, the target setting was oncology wards in acute hospitals including tertiary referral and cancer centres. Studies of non-oncology ward patients were included if the oncology population could be abstracted from a broader study e.g., hospital-wide point prevalence, subset of cancer patients within an index population of older patients with cancer. Studies set in palliative care populations in a “stand alone” inpatient unit or hospice were only included if the setting was combined oncology and palliative care, for example a comprehensive cancer centre. To meet inclusion a delirium assessment with a validated objective tool, or clinical diagnostic criteria was also required.

Studies were excluded if they were solely conducted in the following settings or populations; haematology or non-solid haematological malignancy, non-cancer palliative care, perioperative including surgical oncology, or alcohol withdrawal delirium.

The reason for excluding non-solid haematological malignancy was the consideration that illness trajectories and treatment protocols in this population may differ a great deal from those of solid cancers, similarly for non-cancer palliative care patients. The exclusion of surgical oncology and peri-operative settings was pragmatic as those patients may be admitted to surgical wards with a different background for staff and potentially different delirium aetiologies. Understanding delirium in these patient cohorts is important and we hope that future work will address areas not included in our review as has been the case in recent multicentre delirium prevalence studies [27, 28].

All authors and an academic liaison-librarian were involved in an iterative process to determine search terms. MEDLINE, CINAHL, PsycINFO, EMBASE and SCOPUS databases were searched. Publication date was limited from 1st of January 1996 to 12th of August 2017. A full list of keywords and Medical Sub-heading (MeSH) is available in Appendix 1.

Independent title, abstract, full-text review and cross check was carried out by MBS and IW, using COVIDENCE [29] software, with conflicts resolved by consensus. Where the same study was reported in more than one manuscript, additional information from related or subsequent publications was included where possible [9, 30–34]. Study heterogeneity was not objectively tested, but the overall lower quality of several included studies and issues with reference standards seemed to suggest meta-analysis would not be meaningful, but sources of bias and generalisability were assessed using the Quality Assessment of Diagnostic Accuracy Studies (QUADAS-2) system.[35] Two authors (MBS and IW) independently piloted the QUADAS-2 and subsequently quality considerations and information synthesis was reviewed by all authors consensus was achieved through discussion.

For the purposes of this study, we defined a delirium reference standard as one which determined diagnostic assignment based on an instrument which used an independent reference-rater evaluation [36]. This last point, although identified in the literature was also arrived at via an iterative process that revealed unclear distinctions between screening tools and case ascertainment or diagnostic criteria upon which case identification was verified among included studies. Examples of reference standards in the basis of these criteria are the World Health Organization (WHO) International Classification of Diseases, 10th Revision (ICD-10)[37] or the American Psychiatric Association Diagnostic and Statistical Manual of Mental Disorders (DSM)[1, 38] criteria, applied by a psychiatrist or consultant physician. On the basis of these criteria, the Confusion Assessment Method (CAM) meets reference standard criteria for case ascertainment, only in studies where reference-rater training in the use of the CAM is explicitly-stated [39]. This follows published recommendations for valid use of the CAM[39] along with diagnostic assignment in delirium research [36]. With regard to detection tool we use the term detection instrument (or tool) to include screening tools or other instruments put forward as standardised methods to identify delirium.

With regard to protocol registration, on inception authors were advised that PROSPERO did not currently accept registrations for scoping reviews and was unable to accept our application for protocol registration. The following is an accurate description of our methodology and further information is available on request. The data that support the findings of this study are available from the corresponding author.

## Results

### Search results

The search date was August 12, 2017. Returns were as follows: Medline (211), EMBASE (684), SCOPUS (97), PsycINFO (52) and CINAHL (47). A total of 805 studies were identified with an additional 91 titles added from hand search. Although most duplicates were removed prior, for pragmatic reasons, final removal of duplicates and screening of abstract and date of publication for hand search returns was held over until full-text review. Hand search consisted of hand search of reference lists from included studies as well as search of authors PDF library using delirium as a title search.

**Fig 1:**
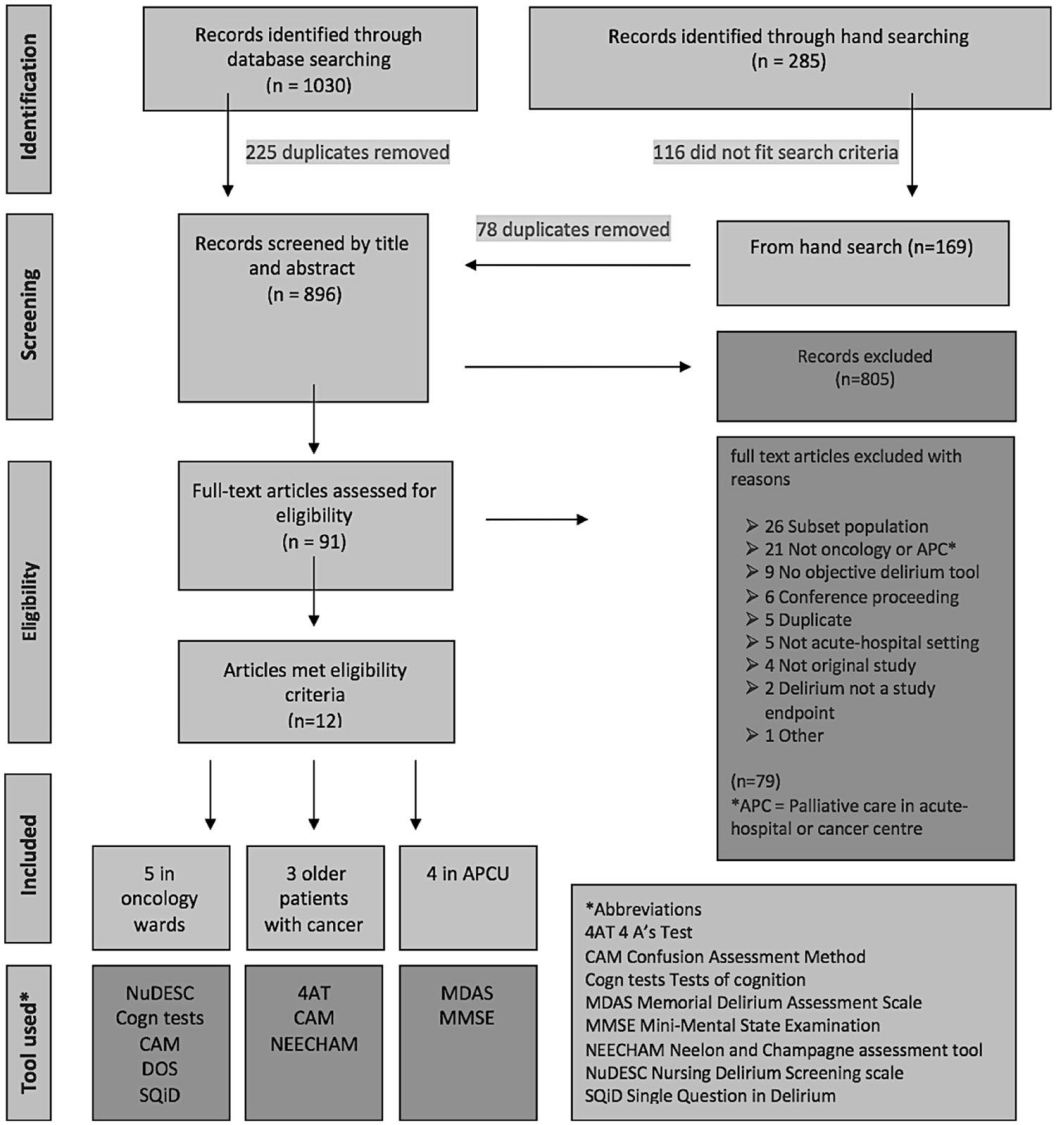
Flow diagram of literature search. Although most duplicates were removed prior, for pragmatic reasons abstract screening for hand search returns was held over until full text review

In total, 91 studies were retained for full-text review, and 12 studies remained for data abstraction and synthesis (Fig. 1)

### Characteristics of included studies

Study recruitment periods ranged from 1997 to 2015. Study design of all 12 studies was observational: six were prospective, six were retrospective. Two studies were secondary analyses of data from prior prospective studies. Tables 1 and 2 provide detailed data for the included studies related to the research questions. Table 3 summarises quality considerations according to QUADAS-2 criteria [35]. Table 4 provides a summary

**Table 1:** Study design and setting

Author (Endnote reference number)	Study setting	Other publications same data set	Study aim	Study period	Patient characteristics, inclusion and exclusion criteria	Total number (of eligible)	Study design flow of participant recruitment/administration of tools
Oncology setting							
Gaudreau et al. JPSM [31]	Haematology, oncology, internal medicine, tertiary hospital Quebec Canada	Gaudreau 2005 September JCO [43]	Determine delirium risk associated with medication exposure	January 21, 2002, to August 4, 2003	Included: admitted, adult, histologic diagnosis of cancer	*n* = 261 (all eligible)	Prospective. Consecutive patients, Nu-DESC incorporated in routine ward care. All patients from admission to discharge for the entire study
Grandahl et al. [40]	Oncology ward, metropolitan cancer centre Denmark	NA	Examine the value of cognitive testing in delirium detection	October 2011–February 2012	Included: admitted adults, histological diagnosis of cancer Excluded: non-Danish speaking. Each participant was included only once. Ward characteristics: patients with cancer who had "complications to their active treatment" or complications to their cancer	*n* = 81 Number of eligible patients not stated	Prospective. Nominated days. Ward staff identified possible cases, then MMSE, CAM, modified mini cog, digit span, and ICD 10. Not stated if consecutive patients or how many eligible patients were excluded from analysis
Ljubisavljevic et al. [3]	Oncology ward metropolitan cancer centre, Australia	NA	Define delirium risk factors	Over 2 periods (ten weeks in total)	Included: admitted, adult, histological diagnosis of cancer. Excluded: inability to undergo interviewing; language barrier; and refusal by the patient, family or physician, admission to a different ward	*n* = 124 (of 156 eligible)	Prospective. All patients during study period were assessed with DOSS on admission. CAM completed nightly for all patients by trained clinical nurses, patients with suspected delirium were reviewed within 24 h to confirm diagnoses of delirium based on DSM iv criteria
Neefjes et al. [41]	Medical oncology ward metropolitan cancer center, Netherlands	NA	Develop delirium prediction model	Jan 1st 2011–June 30th 2012	Included: admitted, adult, solid malignancy	*n* = 574 patients/1733 admissions (all eligible)	Retrospective, All patients. Chart review of DOSS scale outcomes, recorded, twice per week on nominated shifts according to standard hospital procedures. Staff familiar with use of tool
Sands et al. [42]	Medical and radiation oncology ward, comprehensive cancer centre, Australia	NA	Test feasibility of index tool	October 2004–August 2006*	Included: admitted, adults, solid malignancy. Patient or proxy consent. Excluded: unable to complete tests in English	*n* = 19 (of 33)	Prospective. All patients on nominated day approached. Consenting patients were assessed in order of SQiD, MMSE, CAM, MDAS, by one blinded investigator, psychiatrist interview by one of two blinded investigators
Older patients with cancer setting							
Bellelli et al. [27]	108 acute and 12 rehabilitation wards across participating Italian hospitals	NA	To determine the point prevalence of delirium in patients in index population in large multi-centre study	September 30, 2015 all admissions to the participating centers from 00:00 to 23:59	Included: admitted, aged 65 years and older, native Italian speakers, patient or proxy consent. Excluded: coma, aphasia, and end-of-life status. Site recruitment by personal email to the members of four scientific associations (5000 members) 108 acute and 12 rehabilitation wards in Italian hospitals	*n* = 323* (1867 of 2221 eligible in main study)	Prospective. All consenting patients in participating centers from 00:00 to 23:59 of the index day. Data reported here is for patients with cancer diagnosis
Bond et al. Oncology Nursing Forum [32]	General medical wards, 3 tertiary teaching hospitals United States	Bond, S. M. et al. 2008, Cancer Nursing [33]	Determine delirium incidence and risk factors in index population	Not reported in index study, paper with full methodology not found	Secondary analysis of data. Included: admitted, age 65 or older, cancer was main diagnosis or co-morbidity	*n* = 76 Number of eligible patients not stated. Parent study was of 627 hospitalized older adults This was a sub group with cancer	Retrospective. Further methodology not established as original paper not available
Hamaker et al. [44]	Medical or oncology ward. 2 metropolitan academic medical centres and one tertiary teaching hospital, Netherlands	NA	Determine delirium prevalence in index population	November 2002 to March 2006 and April 2006 to March 2008	Included: admitted, age 65 or older. Excluded: too ill, intensive care unit, coronary care unit, or transfer 48 h post admission, unable to speak or understand Dutch	*n* = 292 number eligible not stated	This was a secondary, sub-group analysis of patients with advanced cancer from prospective study. All consenting. Multidisciplinary comprehensive geriatric assessment (CGA) within 48 h of admission. (two medical specialists, a geriatric resident, a clinical nurse specialist, and two research nurses trained in geriatric medicine, who assessed for geriatric conditions including delirium)
Acute palliative care							
de la Cruz, et al. [39]	12-bed acute palliative care inpatient unit in comprehensive cancer centre, USA. (Same centre as Shin 2014 and Mori 2011)	NA	Determine incidence and prevalence of delirium in index population	January 2011 to December 2011	Included: admitted patients	*n* = 609 consecutive patients > 556 total single admissions >	Retrospective. Search of medical records for demographics, ECOG performance status, MDAS score, Edmonton Symptom Assessment Scale (ESAS) score [18], and discharge disposition
Lawlor et al., March, Arch Int Med [9]	14-bed tertiary level Palliative Care Unit in a university affiliated teaching hospital in Canada	Lawlor, P. G. et al. 2000, June, Cancer https://doi.org/10.1001/archinte.160.6.786 [28]	Determine incidence, prevalence, severity and reversibility in index population	February to October 1997	Included: adult, admitted, histological diagnosis of cancer. Excluded unable to speak English fluently, or unable to speak due to tracheostomy	*n* = 104 (of 113 eligible)	Prospective. Consecutive admissions, verbal consent, MMSE on admission and twice weekly. If MMSE threshold reached, DSM diagnosis by palliative care physician. If delirious then MDAS to assess severity and progress
Mori et al. [47]	12 bed acute palliative care inpatient unit in comprehensive cancer centre, USA. (Same centre as Shin de la Cruz)		Determine the influence of delirium severity and survival	June 2006 to December 2007	Included: admitted, adult, advanced cancer. Admissions from emergency centre (EC) and outpatient clinic with ESAS data from within 24 h of APCU admission (baseline) and 3 to 5 days (follow-up) of APCU admission were included. Excluded: transfers from oncology ward excluded, missing symptom assessment score, early death or discharge	*n* = 166 (of 181 eligible)	Retrospective. Consecutive patients. In some patients, the ESAS was not completed because of the diagnosis of delirium. In such cases, other information was collected and included in analysis. Excluded patients who died before third day of APCU admission were excluded
Shin et al. [48]	Acute palliative care inpatient unit in comprehensive cancer centre, USA (same and Mori and de la Cruz)			September 1, 2003 and August 31, 2008	Index group: Emergency centre (EC) admissions Comparator group: inpatient (IP) transfers from oncology ward	*n* = 610 (of 612 eligible)	Retrospective. Institution's database identified 2568 MDAS scores data. Unclear how many unique patients represented by these scores. Data abstracted from electronic record for patients admitted from EC or oncology ward transfers

^*^Unpublished data:,*4AT* 4 A’s Test, *Nu-DESC* Nursing delirium screening scale, *MMSE* mini-mental state exam, *CAM* Confusion Assessment Method, *ICD* *10* international classification of diseases 10th revision, *DOS* The Delirium Observation Screening scale, *SQID* Single Question in Delirium, *MMSE* Mini-mental state exam, *MDAS* Memorial Delirium Assessment Scale, *CGA* comprehensive geriatric assessment, *ICD-10* international classification of diseases 10th revision, *MMSE* mini-mental state exam, *ECOG* Eastern Cooperative Oncology Group performance status, *MDAS* Memorial Delirium Assessment Scale, *ESAS* Edmonton Symptom Assessment Score, *APCU* Acute Palliative Care Unit

**Table 2:** Patient Characteristics, Study Tools and Delirium Rates

Author (endnote reference number)	Cancer primary site (%)	Age in yrs, mean sd (range) mlos (days)	Other correlates of burden of disease	Index delirium tool assessor sensitivity and specificity vs diagnostic standard	Other delirium detection tools	Diagnostic or research reference standard, assessor, assessor training	Delirium rate test reversibility
Oncology setting							
Gaudreau et al. JPSM [31]	Hematologic 86(33%) Gastrointestinal tract 35(13.4%) Lung 21(17%) Bones/soft tissue 24 (9.2%) Genital 11(4.2%) Urinary 14(5.4%) Breast 16 (6.1%) Ovary 12(4.6%) Colorectal 26(10%) Other 16(6.1)	59.6 ± 14.3	154/261 (59%) loco regional disease only	TOOL: NuDESC ASSESSOR: routine administration by bedside nurses familiar with tool. Sensitivity: 0.857 (0.654–0.950) Specificity: 0.868 (0.727–0.943)	1. CAM assessed by psychiatrist (73% of patients) 2. MDAS by research nurse 3. MDAS by psychiatrist 4. DSM-IV by research nurse 5. DSM-IV by psychiatrist CAM training not specified	TOOL: CAM; ASSESSOR: research nurse; ASSESSOR TRAINING: research nurses were trained over six 2-h on- site sessions with psychiatrists in the use of the CAM, the MDAS, and the DSM-IV criteria for delirium. Inter-rater reliability: kappa = 0.89 (95% CI, 0.75–1.0) of research nurse—psychiatrist for the CAM	Incidence 16.5% (43/261) on basis of NuDESC REVERSIBILITY: not reported
Grandahl et al. [40]	Gastrointestinal 30 (37%), Lung 28 (35%), Breast 16 (20%) Other 7 (9%)	68.5 ± 7.8 (42—86)	not reported	Battery of tests of cognition	CAM training not specified	TOOL: ICD 10 diagnosis ASSESSOR: not stated ASSESSOR TRAINING: not stated	Prevalence 33% (27/81) on basis of DSM IV REVERSIBILITY: not reported
Ljubisavljevic et al. [3]	Haematological 70 (57%) gastro-oesophageal 23 (19%) breast 11 (9%) melanoma, osteogenic sarcoma, germ cell tumour 4 (3%) each, colon 3 (2%), other 3 (2%)	53 -SD and range not reported mean LOS 5	CNS tumour 9%	NA	CAM by psychiatrist for positive cases. Training not specified. Clinical review by consultant psychiatrist for all positive cases and a sample of 10 (consenting) negative cases	TOOL: CAM ASSESSOR: ward nursing staff ASSESSOR TRAINING: weekly sessions prior to and throughout study period CAM completion 80%	Prevalence 18% (26/145 admissions) REVERSIBILITY: not reported
Neefjes et al. [41]	Gastrointestinal 196 (34%) Genito-urethral 22 (4%) Head and Neck 19 (3%) Breast 9 (2%) Lung < 1	60 ± 13.1 MLOS 3 (IQR 2–6)	Included: acute admission (42%) median ECOG 1, alive at discharge 96% 81% "disseminated cancer" 14/81 CNS metastases	TOOL: DOSS or clinical diagnosis, and without rejection of delirium in the notes ASSESSOR: clinical nurses as part of routine care, or clinician diagnosis	NA	NA	Incidence 3.5% all admissions 7.8% (57/730) for un-scheduled admissions on basis of DOSS REVERSIBILITY: not reported
Sands et al. [42]	Breast 3/18, lung 2/18 prostate 2/18. 6/18 other, unknown 3/18 *	53 ± 14.3 (30–79)*	5/19 distant metastases*	Single question in delirium (SQiD), novel tool	CAM administered by medical students training not specified	TOOL: DSM IV criteria ASSESSOR: Psychiatrist, clinical diagnosis ASSESSOR TRAINING: [core professional competence]	Prevalence 27% (5/18) on basis of DSM REVERSIBILITY: not reported
Older patients with cancer							
Bellelli et al. [27]	NA	81.2 ± 7.5*	Charlson comorbidity index 5.3 + 2.1, Katz's ADL 3.8 + 2.3 Comorbid dementia 53 (16.4)	TOOL 4AT ASSESSOR: attending physician	NA	NA	OLDER CANCER Point prevalence 19.2% (62/323) on basis of 4AT REVERSIBILITY: not reported
Bond et al. Oncology Nursing Forum[32]	Multiple myeloma 13 (17%), Lymphoma 6 (8%), Lung cancer 11 (15%), prostate cancer 11 (15%), breast cancer 8 (11%) Other 27 (36%)	74.4 ± 7.29 (65–96) Mean LOS 9.8	APACHE II score 14.9 (moderate illness severity). IADL score of 6.8	TOOL: NEECHAM ASSESSOR and TRAINING: unable to access primary source referenced	NA	NA	OLDER CANCER Prevalence 57% (43/76) on basis of NEECHAM REVERSIBILITY: 13/43 (30%)
Hamaker et al. [44]	Leukaemia 12 (4%), Pancreatic 36 (12%), Colon 32 (11%), Oesophageal 26 (9%), Cholangiocarcinoma 23 (8%), Lymphoma 21 (7%), Breast 18 (6%), Lung 18 (65), Prostate 16 (5.5%), Stomach 15 (5%), Bladder 14 (5%)	74.9 (65.0–96.2) MLOS 8 (1–80)	48% receiving supportive care only 55% receiving active[antitumour] treatment 95% living independently 43% metastatic disease at inclusion. 77% impaired ADL. Mean Charlson co-morbidity score 1.1. 15% (31/201) Global cognitive impairment	NA	NA	TOOL: CAM ASSESSOR: "nurse" ASSESSOR TRAINING: not stated	OLDER CANCER Prevalence 21.5% (61/283) On basis of CGA incorporating CAM REVERSIBILITY: not reported
Acute palliative care setting							
de la Cruz, et al. [22, 46]	Haematological 74(13%), solid tumour 382 (86%)	56.51 ± 13.85	182 (32%) died index admission ECOG > or = to 3 508/556 (91%)	TOOL: MDAS cutoff ASSESSOR: daily routine, palliative care physician	TOOL: DSM IV ASSESSOR: palliative care physician. Number assessed unclear	NA	APCU Point prevalence on admission 71% 229/556 Incidence: 16.9% 94/327 REVERSIBILITY: 26% (68/229)
Lawlor et al. 2000, March, Arch Int Med [9]	Lung 17 (30.4%), genitourinary 16 (28.6%), breast in 8 (14.3%), gastrointestinal in 7 (12.5%), haematologic in 4 (7.1%), head and neck in 3 (5.3%), and other in 1 (1.8%)	64.14 ± 10	distant mets: 86/104 (83%)	TOOLS: MMSE with cutoff (assessor not explicit)	TOOL: MDAS if DSM positive	TOOL: DSM IV (not applied to all participants) ASSESSOR: palliative care physician ASSESSOR TRAINING: not stated	APCU Point prevalence on admission 42% (44/104) incidence 45% (27/60) on basis of MMSE plus MDAS with cutoff REVERSIBILITY: 46/94 (49%)
Mori et al. [47]	Gastrointestinal 47 (28%) Lung 33 (20%) Breast 10 (6%) Haematological 11 (7%) Gynaecological 10 (6%) Head and Neck 9 (5%) Urological 23 (14%) Other 23 (14%)	59 ± 13 (Patients who died) 61.3 ± 14.4 (patients alive at discharge) MLOS 8 days (4–12)	metastases 89%	TOOL: MDAS ASSESSOR: daily routine, palliative care physician or clinical judgment of palliative care physicians, advanced practice nurses, or palliative care clinic nurses		NA	APCU Prevalence 73/166 43% on basis of MDAS cutoff REVERSIBILITY: not reported
Shin et al. [48]	Haematological 58 (10%) Gastrointestinal 129 (22%) Respiratory 149 (25%) Breast 42 (7%) Genitourinary/gynaecological 85 (14%) Head and Neck 41 (7%) Others 96 (16%)	58.9 (95% CI 57.8–60.0) MLOS (in APCU) 8.0 (7.6–8.4)		TOOL MDAS or clinical diagnosis ASSESSOR: daily routine, palliative care physician PURPOSE: to determine influence of symptoms on survival		NA	APCU Period prevalence: 48% (284/610) on basis of MDAS cutoff REVERSIBILITY: not reported

*MLOS* median length of stay *Unpublished data, *ECOG* Eastern Co-operative Oncology Group performance status, *CAM* Confusion Assessment Method, *MDAS* Memorial Delirium Assessment Scale, *MMSE* Mini-mental state exam, *DSMIV* Diagnostic and Statistics Manual 4th edition, *ICD-10* International Classification of diseases 10th version, *for cancer patient subset personal communication, *4AT*: 4 A's delirium assessment test, *NEECHAM* Neeson and Champagne confusion Confusion Scale, *CAM* Confusion Assessment Method, *APACHE* *II* Acute Physiology and Chronic Health Evaluation II Score, *ECOG* Eastern collaborative oncology group performance status, *ADL* Activity of Daily Living, *CGA* Comprehensive Gerriatric Assessment, *MMSE* mini-mental state exam, *MDAS* Memorial Delirium Assessment Scale, *ESAS* Edmonton Symptom Assessment Score, *APCU* Acute Palliative Care Unit

**Table 3:** Quality assessment using QUADAS tool

Author (Endnote reference number)	Risk of bias patient selection	Risk of bias index test	Risk of bias reference standard	Risk of bias flow and timing	Generalisability patient selection	Generalisability index test	Generalisability reference standard
Oncology setting							
Gaudreau et al. April [31]	Low risk	Low risk	Low risk	Low risk	Intermediate risk	Low risk	Low risk
Grandahl et al. [40]	Low risk	Intermediate risk	Intermediate risk	Intermediate risk	Intermediate risk	Low risk	Low risk
Ljubisavljevic et al. [3]	Low risk	Low risk	Low risk	Low risk	Low risk	Low risk	Low risk
Neefjes et al. [41]	Low risk	Low risk	Not used	Intermediate risk	Intermediate risk	Low risk	Not used
Sands et al. [42]	Low risk	Intermediate risk	Low risk	Low risk	Intermediate risk	Low risk	Low risk
Older patients with cancer setting							
Bellelli et al. [27]	Low risk	Low risk	Low risk	Low risk	Intermediate risk (for cancer subset)	Low risk	Low risk
Hamaker et al. [44]	Low risk	Not used	Low risk	Low risk	Low risk	Low risk	Low risk
Bond et al. [32]	Insufficient information to assess						
Acute palliative care setting							
de la Cruz, et al. [22, 46]	Intermediate risk	Higher risk	Intermediate risk	Intermediate risk	Intermediate risk	Intermediate risk	Higher risk
Lawlor et al. [9]	Low risk	Higher risk	Low risk	Higher risk	Low risk	Higher risk	Higher risk
Mori et al. 2011 [47]	Intermediate risk	Higher risk	Higher risk	Intermediate risk	Intermediate risk	Higher risk	Higher risk
Shin et al. [48]	Intermediate risk	Higher risk	Higher risk	Intermediate risk	Intermediate risk	Intermediate risk	Higher risk

Total number of studies in categories: Study Setting: Oncology (5), older patients with cancer (3), acute palliative care (4). Diagnostic reference standards (2): DSM Diagnostic and Statistics Manual (various editions ICD-10 International Classification of Diseases (10th version) and CAM by trained operator (1). Tools used for delirium detection: MDAS (4), CAM (3), DOSS (1), Cognition testing (1), 4AT (1), NEECHAM (1), NuDESCC (1) Note: (total greater than number of studies as one study used two methods)

**Table 4:** Delirium Rate by study and tool used

Author	Age in yrs, mean sd (range if reported)	Delirium assessment	Delirium rate recruitment consecutive or non consecutive admissions
Oncology inpatients			
Gaudreau et al. [31]	59.6 ± 14.3	NuDESC	Incidence 16.5% Consecutive
Grandahl et al. [40]	68.5 ± 7.8 (42—86)	DSM IV	Prevalence 33% Non-consecutive
Ljubisavljevic et al.[3]	53	NA	Prevalence 18% Consecutive
Neefjes et al. [41]	60 ± 13.1	TOOL: DOSS or clinical diagnosis	Incidence 3.5% all admissions 7.8% (57/730) un-scheduled admissions Consecutive
Sands et al. [42]	53 ± 14.3 (30–79)*	DSM IV/DSM IVR	Prevalence 27% (5/18) Non-consecutive
Older patients with cancer			
Bellelli et al. [27]	81.2 ± 7.5*	TOOL 4AT	Point prevalence 19.2% (62/323) Consecutive
Bond et al. [32]	74.4 ± 7.29 (65–96)	NEECHAM	Prevalence 57% (43/76) Non-consecutive
Hamaker et al. [44]	74.9 (65.0–96.2)	GCA	Prevalence 21.5% (61/283) Consecutive
Acute palliative care setting			
de la Cruz et al. [22, 46]	56.51 ± 13.85	MDAS	Point prevalence on admission 71% 229/556 Incidence: 16.9% 94/327 Consecutive
Lawlor et al. [9]	64.14 ± 10	MMSE	Point prevalence on admission 42% (44/104) incidence 45% (27/60) Consecutive
Mori et al. [47]	59 ± 13 (Patients who died) 61.3 ± 14.4 (patients alive at discharge)	MDAS	Prevalence 73/166 43% Consecutive
Shin et al. y[48]	58.9 (95% CI 57.8–60.0)	MDAS or clinical diagnosis	Period prevalence: 48% (284/610)

*4AT* 4 As test, *Nu-DESC* Nursing delirium screening scale, *MMSE* mini-mental state exam, *CAM* Confusion Assessment Method, *GCA* Comprehensive Geriatric Assessment, *ICD 10* international classification of diseases 10th revision, *DOS* The Delirium Observation Screening scale, *MDAS* Memorial Delirium Assessment Scale

Studies were grouped into three categories on the basis of the clinical setting: (1) inpatient, acute-hospital or comprehensive cancer centre oncology ward; (2) older oncology patients (patients > 65 years, admitted to acute hospitals under any admitting team, with cancer as the primary diagnosis or co-morbidity); and (3) palliative care ward in acute-hospital or comprehensive cancer centre (APCU). The rationale for this grouping was based in the observation that clinical care for oncology patients occurs largely in one of these three settings, but that from the point of view of research, these settings tend to be studied independently; we also wanted to decrease heterogeneity within subgroups, but facilitate understanding the use of delirium detection tools across the spectrum of admitted adult patients with cancer to improve care in this cohort.

Five studies [3, 31, 40–42] were in the adult-oncology setting. Three of these approached all patients on nominated days [40–42], while two studies approached all admitted patients [3, 43]. Three studies were of older cancer patients [27, 32, 44]. One of these was a point prevalence study in which patients over 65 years were recruited from more than 100 hospitals across several regions of Italy during one 24-h period [27]. Unpublished subset data on patients in this last cohort, were provided by the author. (personal communication G Bellelli, October 2017) [45] A further study recruited all patients aged 65 years or older admitted to the general medicine or oncology ward in two Dutch teaching hospitals [44]. The remaining study in older oncology patients, was a secondary analysis of a subset of cancer patients from a previous study, composed of patients from three North American centres [32]. Four studies [9, 46–48] were in an APCU. Three of these [46–48] were retrospective and based in the same health care facility.

Three studies focused on patients with cancer referred to consultation psychiatry services and reported misdiagnosis of neuropsychiatric conditions, with two studies reporting a missed diagnosis of delirium in 46%, and a further study reporting 63% missed cases [11, 13, 49] .

### Patient recruitment and demographics

Patient characteristics were described in varying detail: four studies [3, 9, 30, 40] specified histological diagnosis of cancer, and three specified consecutive recruitment [27, 34, 46]. Four studies gave a detailed description of recruitment [3, 44, 50], and seven provided the number of eligible patients when providing number of participants. Eight studies [3, 9, 31, 41, 42, 46–48] presented flow diagrams or data accounting for eligible patients not included in recruitment or analysis.

Ten of 12 studies reported primary cancer types. All reported age; the average of the mean age (years) in each setting were as follows: oncology 59, older cancer 78, and APCU 60. Six studies reported length of hospital stay [3, 32, 41, 44, 47, 48]; these were reported as mean or median, and ranged from 3 to 9.8 days. Clinical information describing cancer stage, co-morbidity burden, overall illness severity, functional status or vital status at discharge, were not uniformly described. Only one of five studies in the adult oncology setting provided detailed information that described markers of burden of disease [41]. Six of 12 studies across all setting subgroups reported the stage of cancer in terms of metastatic versus loco-regional disease [9, 41–44, 47]. One study reported the number of patients receiving anti-cancer treatment [44].

### Scoping questions; data relating to our four research questions

1. Which instruments are most commonly used to detect delirium?

Of the studies meeting our inclusion criteria, five used previously validated instruments for clinical detection of delirium: Nursing Delirium Screening scale (NuDESC, *n* = 1); Delirium Observational Screening Scale (DOSS, *n* = 1); four A’s test (4AT, *n* = 1); Neelon and Champagne (NEECHAM, *n* = 1); and, Memorial Delirium Assessment Scale (MDAS, *n* = 4). One study tested a novel delirium screening tool (Single Question in Delirium; SQiD) and one tested cognitive measures (Clock Drawing Test, Mini Cognitive, Digit Span Test) against a reference standard. Six studies included a second delirium detection tool, as presented in Table 2.

2. Which reference standards have been used to measure rates of delirium?

Four of 12 studies met criteria for a delirium reference standard for case ascertainment [36]. Two studies in the adult oncology setting used diagnostic criteria, namely the ICD 10 [40] (assessor characteristics were not stated), and DSM IV/IVR (assessed by final year psychiatry fellow or psychiatrist) [42]. Two studies used the CAM in a way that met criteria for use as a reference standard, including an account of assessor training [3, 31] .

Seven studies used a screening tool alone as the basis of case ascertainment of delirium: MDAS (*n* = 4); CAM (*n* = 2); DOSS (*n* = 1); 4AT (*n* = 1); NEECHAM (*n* = 1); Nu-DESC (*n* = 1), and one used a battery of tests of cognition (*n* = 1). Neither of these two studies using the CAM as the basis of delirium case ascertainment, specified assessor training [40, 44]. Of the prospective studies, Bellelli et al. used the 4AT assessed by the attending physician [27]; Lawlor et al. used DSM IV to confirm participants who had Mini-Mental State Exam (MMSE) scores above a cut-off point on first-line testing [9]; and Gaudreau et al. used the Nu-DESC, applied by trained bedside nurses familiar with this tool [43]. Of the retrospective studies, Neefjes et al. used the DOSS applied by trained bedside nurses familiar with the tool [41], and three studies used a cut-off score on the MDAS to identify delirium cases on chart review [46–48]. Studies comparing MDAS, MMSE, 4AT, NEECHAM tools for detection in clinical practice compared to a reference standard were not identified in our target settings, so it is not possible to ascertain the rate of missed delirium from the available literature.

3. What is the incidence and prevalence of delirium in this setting?

Rates of delirium incidence and prevalence reported by studies in this review are presented in Table 2. Table 4 presents a summary of tools used and delirium rates established on that basis. Consecutive or non-consecutive recruitment is also reported to aid interpretation of delirium rates.

In the adult oncology setting, Neefjes et al. found a delirium incidence of 3.5 per 100 admissions or 7.8 per 100 of unscheduled admissions [41], and Gaudreau et al. reported an incidence of 16.5% [43]. Three studies in this sub-setting presented prevalence data; 18% [3], 27% [42], and 33%[40] respectively. In the APCU sub-population, prevalence rates of 42% [9], 43%,[47], 48% [48], and 58%[46] were found. The three studies of older cancer patients found prevalence rates of 19.2% [27], 21.5% [44], and 57%,[32], respectively. One study in the oncology sub-setting [41], and one in the APCU sub-setting [46], reported the frequency of delirium subtypes: hyperactive 11/52 (21%) and 61/246 (25%); hypoactive 20/52 (38%) and 73/245 (30%); mixed 18/52 (35%) and 112/246 (46%); and not known 3/52 (6%) and 73/246 (31%) respectively.

4. What is the rate of reversibility of delirium in this setting?

Delirium reversibility was reported in three studies, two of these were in APCU settings; 46/94 (49%) [9], 68/229 (26%) [46], and one in older patients with cancer 13/43 (30%)[33]. Of four studies reporting reversibility [9, 32, 46, 47], two[9, 32] did not explicitly state how reversibility was defined; one used the MDAS or clinical documentation to determine delirium reversibility and another used a MDAS cut off score at day five. Although one of these[47] referenced a predating publication, this could not be found. The other three used MDAS scores collected as part of routine clinical care to define reversibility. Bond et al. found that patients with fewer precipitating factors were more likely to have a resolution but found only prior cognitive impairment to be negatively associated with delirium reversal [33].

In the Lawlor study delirium associated with opioids and non-opioid psychoactive medication and dehydration were more likely to be reversed while non-reversed delirium was more common when associated with a respiratory infection, pulmonary cancer and metabolic causes [9].

## Discussion

We identified significant knowledge gaps regarding epidemiological characteristics of delirium in oncology inpatients. A variety of delirium screening tools were identified, but few studies used accepted diagnostic or reference standards for case ascertainment. Sources of bias included study design and generalisability. A small number of eligible studies reported reversibility of delirium.

Delirium is a multifactorial syndrome. The relationship of delirium risk with demographic factors such as age and clinical factors (e.g. cancer diagnosis), is complex. One of the studies in the older cancer sub-group provided comparative figures of delirium point prevalence in cancer and non-cancer patients; 19.2% (*n* = 323) for patients with cancer compared to 23.9% (*n* = 1544) of the patients with no cancer diagnosis (*p* = 0.06) [27]. Within the included studies, but also in non-cancer settings reported elsewhere, multiple delirium risk factors such as co-morbidities, presence of physiological disturbance and medication burden are factors that seem to be constants in understanding overall delirium risk. [7, 9, 13, 41, 43, 51–59]

Delirium screening tools have been developed, and validated, predominantly in older, hospitalised patients [60–64]. The tools for which psychometric properties have been assessed in cancer in-patients in the acute setting, are the Nu-DESC and MDAS [31, 34, 65]. There is a clear rationale for use of the Nu-DESC as a delirium screening tool [31]. The 4AT has been tested in “stand-alone” palliative care, inpatient settings[66] and for older adults admitted to hospital [50, 67, 68]. The MMSE can be used to screen for cognitive impairment it has been found to have poor performance as a bedside tool for identifying delirium [69, 70].

The Confusion Assessment Method [36] (CAM) has several versions [67], and has well-established psychometric properties [61]. In the main, studies have supported the use of the CAM for delirium screening in research settings providing there is strict adherence to operator training, however, one study suggests even in the context of strict adherence to CAM training, sensitivity of the CAM may not be sustained [68]. Our review found that most studies using the CAM for case ascertainment did not describe the training staff underwent [3, 37, 39, 41] and one, described difficulty in attaining adequate training in a clinically embedded research context [42, 71].

Four of the twelve included studies used the MDAS as a basis for case confirmation of delirium [9, 46–48]. The MDAS was designed specifically to rate delirium severity[65], it has face validity and uptake, further formal validation studies for its use as a delirium screening tool would build on the existing psychometric data and help to reinforce the attributes of the tools [64]. One perspective looks at the balance between the positive features of usability of the MDAS compared with some other tools, and the effect of the breakdown and operationalisation of delirium features within the MDAS which does not support the syndromic nature of delirium diagnosis in terms of coexisting core features. Although it identifies delirium symptoms, regardless of the cut-off score specified to identify delirium, the MDAS risks false positive results, as patients with delirium symptoms who do not fit the core diagnostic criteria for syndromic delirium as characterised by coexistent core features may be labelled case positive. Several studies in this review used the MDAS alone for case ascertainment, which may bias reported detection rates [48, 64].

Clinical operationalisation appeared to be the major driver of choice of delirium screening tools. Delirium diagnosis is complex, multidimensional and not intuitive for bedside staff. DSM 5 criteria require 5 characteristics and so while screening tools may gain in usability through operationalisation they lose precise application of the necessarily coexistent core features that define delirium. In the research setting, we found clear demarcation between the index tool and the chosen reference standard was not always evident. A blurring of the distinction between screening tools and diagnostic reference standards used for case confirmation for validation purposes was found. More specifically where references standards were other than DSM or ICD based reporting of reference-rater training was at times lacking. The importance of tool selection to fit the intended purpose is an important finding of our review.

In APCUs delirium rates were higher than in oncology inpatients but given methodological constraints in studies within this setting, results may not be representative. The use of the MDAS may have contributed to inflated delirium rates reflecting the way the tool is operationalised. In the older cancer patient cohorts, differences in delirium incidence and prevalence might be accounted for by study heterogeneity and patient recruitment. This is an important issue for future work, as establishing delirium incidence and prevalence in inpatient oncology settings is an important step in management. Better understanding of how to use available tools will improve management and inform education initiatives in this setting.

Criteria for delirium reversal were inadequately defined in studies, making it difficult to compare delirium reversibility across studies. These data may be further constrained by retrospective methodology, the absence of a diagnostic reference standard, or study flow reliant on clinical documentation. Ascertainment of delirium reversibility requires prospective, longitudinal study design, use of a robust diagnostic standard and explicit definition of delirium reversal. Assessment of delirium reversibility is an important issue for consideration in the design of future studies.

Patient selection, choice of the delirium screening tool and the choice of the diagnostic reference standard, were all identified as a source of bias on QUADAS-2 criteria [35]. Recruitment flow was also an important consideration. For example, patient selection methods at times risked exclusion of potentially delirious patients due to retrospective design, convenience sampling, and ascertainment bias.

Adherence to consensus recommendations for reporting patient characteristics and wherever possible the use of assessment tools and delirium reference standards will improve epidemiological studies of delirium in this setting [36, 72, 73] .

Limitations to our review include those related to the methodology of the original studies as well as a limitation to the English language. The search was limited to publications between 1996 and 2017. The discussion has aimed to identify recent updates in the area, again these are largely limited to aged care or stand-alone settings, with one systematic review of delirium in palliative care fining an incidence of 9–57% across hospital palliative care consultative services, with a majority of patients having cancer diagnoses [20]. a further systematic review, again in the palliative care setting, identified 14 delirium detection tools and heterogeneity of methods [23], Important questions for future work include which tools translate well to inpatient oncology from aged care and stand-alone inpatient palliative care settings, which tools are most suitable for patients, carers and staff, and which reference standards are most appropriate. Requirements for clinical and research uses of detection tools will differ according to purpose, however establishing methodical approaches to the detection of delirium in either setting is a prerequisite to determining the incidence, prevalence and reversibility of delirium for oncology inpatients. Maintaining a clear accountability for the validation and purpose of the tool, and its psychometric characteristics when applying it to clinical screening/detection is critical as is the requirement in research uses to select a reference standard with established reference-rater methodology, is extremely important.

Choosing a tool for delirium detection in the clinical oncology setting will vary according to operational issues such as staff training and preference, however, it is important that tools are fit for purpose, and where possible, have been validated in the same clinical setting. While patient profiles may be similar across palliative care, aged care and some oncology inpatient settings, staff competencies will be more specifically related to setting. Delirium detection and diagnosis must be a core competency for clinical teams in acute settings, however, operational characteristics may render a tool selection may vary according to operational setting, the exact tool chosen is not as important as the review of characteristics that makes it fit for purpose/setting.

Our review, found gaps in the validation of tools in for use in oncology inpatients. At present extrapolation from findings in other acute hospital settings, such as aged care, may help support a more robust selection for this population for the time being. As further validation occurs in acute oncology settings the evidence base for selection tools to detect the presence or resolution of delirium in this clinical setting should improve.

The knowledge gaps identified to generate new hypotheses for future investigation. We recommend the optimal description of patient characteristics, selection of delirium detection tools appropriate to the setting, use of reproducible methods of patient selection and diagnostic assignment using a reference standard with appropriate reference rater methodology. Our results indicate that a determination of the incidence, prevalence, and reversibility of delirium in the inpatient cancer population is both lacking and overdue. Addressing these knowledge gaps will help to provide a more robust evidence base to inform ongoing efforts for effective prevention, detection and management of delirium in the inpatient oncology setting.

## Supplementary Information

Below is the link to the electronic supplementary material.

Supplementary file1 (DOCX 120 KB)

Supplementary file2 (DOCX 1050 KB)

Supplementary file3 (DOCX 14 KB)

## Declarations

### Availability of data and material

Upon request.
