# Supplementary material for: The detection of delirium in admitted oncology patients: a scoping review
Source: Eur Geriatr Med. 2022 Jan 15;13(1):33–51. doi: 10.1007/s41999-021-00586-1 (PMC8860783; doi:10.1007/s41999-021-00586-1)
Supplement: Supplementary file 1 — Supplementary file1 (DOCX 120 KB) [file 41999_2021_586_MOESM1_ESM.docx]

**Preferred Reporting Items for Systematic reviews and Meta-Analyses extension for Scoping Reviews (PRISMA-ScR) Checklist**

| **SECTION** | **ITEM** | **PRISMA-ScR CHECKLIST ITEM** | **REPORTED ON PAGE #** |
| --- | --- | --- | --- |
| **TITLE** | | | |
| Title | 1 | Identify the report as a scoping review. | Click here to enter text. |
| **ABSTRACT** | | | |
| Structured summary | 2 | Provide a structured summary that includes (as applicable): background, objectives, eligibility criteria, sources of evidence, charting methods, results, and conclusions that relate to the review questions and objectives. | BACKGROUND: Delirium is a frequent and serious presentation for cancer patients. Here we review delirium detection in hospitalized adult oncology settings. PATIENTS AND METHODS: MEDLINE, EMBASE, CINAHL, PsycINFO, and SCOPUS databases were searched from January,1996 to August, 2017. Key concepts are: delirium, cancer, in-patient oncology and delirium screening/detection. RESULTS:  Of 896 unique studies identified; 91 met full text review criteria. Of 12 eligible studies, four applied recommended case ascertainment to all patients, three used delirium screening tools alone or with case ascertainment tools sub-optimally applied, four used tools not recommended for delirium screening or case ascertainment, one used the Confusion Assessment Method with insufficient information to determine if it met case ascertainment status. Two studies presented delirium incidence rates; 7.8%, and 17% respectively. Prevalence rates ranged from 18-33% for general medical or oncology wards; 42-58% for Acute Palliative Care Units (APCU); and for older cancer patients: 22% and 57%. Three studies reported reversibility; 26% and 49% respectively (APCUs) and 30% (older patients with cancer). Six studies had low risk of bias according to QUADAS criteria; all studies in the APCU setting were rated at higher risk of bias. Tool selection, study flow and recruitment bias reduced study quality. CONCLUSION: The knowledge base for improved interventions and clinical care in adult oncology is limited by the low number of studies. A clearer distinction between screening and diagnostic tools is required to provide an improved understanding of the rates of delirium and its reversibility in this population. |
| **INTRODUCTION** | | | |
| Rationale | 3 | Describe the rationale for the review in the context of what is already known. Explain why the review questions/objectives lend themselves to a scoping review approach. | The incidence of delirium in hospitalized cancer patients is not well characterized. Initial searches identified data from inpatient palliative care but few studies included all cancer inpatients. As there are some similarities and differences across various settings and age groups we wished to understand the evidence supporting epidemiological information regarding delirium incidence, prevalence and reversibility in 3 cancer inpatient settings 1. Older general hospital cohorts with an identifiable subset of patients with cancer 2. Inpatient oncology wards 3. Dedicated palliative care units within cancer centres or general hospitals (but excluding stand-alone Hospice or palliative care inpatient units) |
| Objectives | 4 | Provide an explicit statement of the questions and objectives being addressed with reference to their key elements (e.g., population or participants, concepts, and context) or other relevant key elements used to conceptualize the review questions and/or objectives. | 1. Which instruments are most commonly used to detect delirium?  2. Which reference standards have been used to measure rates of delirium and/or compare performance of delirium screening instruments?  3. What is the incidence and/or prevalence of delirium in this setting? and  4. What is the rate of reversibility of delirium in this setting?  Our search strategy centred on four key domains; delirium, cancer, in-patient oncology, and delirium detection. Full inclusion criteria are: original study, English language, focus of the study must be delirium (e.g. not: confusion, cognitive impairment, acute brain syndrome), and setting must be oncology with a focus on adults in the oncology setting. The target setting was oncology wards in acute hospitals including tertiary referral and cancer centres. (Emergency departments fit inclusion if the focus was oncology and the patient was admitted). Non-oncology ward patients were included if the oncology population could be abstracted from a broader study (e.g., hospital-wide point prevalence, subset of cancer patients within an index population of older patients with cancer). Palliative care patients in a “stand alone” in-patient unit or hospice were only included if admitted to a combined oncology or palliative care setting, for example a comprehensive cancer centre. To meet inclusion a delirium assessment with an objective tool, or clinical diagnostic criteria was also required.  Studies were excluded if they were solely conducted in the following settings or populations; haematology or non-solid haematological malignancy, non-cancer palliative care, perioperative including surgical oncology, or alcohol withdrawal delirium. |
| **METHODS** | | | |
| Protocol and registration | 5 | Indicate whether a review protocol exists; state if and where it can be accessed (e.g., a Web address); and if available, provide registration information, including the registration number. | A review protocol exists and is available on request. PROSPERO does not currently accept registrations for scoping reviews and was unable to accept our application for protocol registration.  The following is an accurate description of our methodology and further information is available on request. The data that support the findings of this study are available from the corresponding author. |
| Eligibility criteria | 6 | Specify characteristics of the sources of evidence used as eligibility criteria (e.g., years considered, language, and publication status), and provide a rationale. | All authors and an academic liaison-librarian were involved in an iterative process to determine search terms. MEDLINE, CINAHL, PsycINFO, EMBASE and SCOPUS databases were searched. Publication date was limited from 1st of January 1996 to 12th of August 2017. A full list of key words and Medical Sub-heading (MeSH) is available in **Appendix 1.**  Independent title, abstract, full text review and cross check was carried out by MBS and IW, with conflicts resolved by consensus. Where the same study was reported in more than one manuscript, additional information from related or subsequent publications was included where possible. Study heterogeneity precluded meta-analysis but sources of bias and generalisability were assessed using the Quality Assessment of Diagnostic Accuracy Studies (QUADAS) system.^26^ Quality considerations and information synthesis was undertaken by all authors.  For the purposes of this study we defined a delirium reference standard as a diagnostic assignment tool such as the World Health Organization (WHO) International Classification of Diseases, 10th Revision (ICD-10) or the American Psychiatric Association Diagnostic and Statistical Manual of Mental Disorders (DSM)^1,28^ criteria, applied by a psychiatrist or consultant physician. The Confusion Assessment Method (CAM) was included as a reference standard for case ascertainment for the purposes of this review, only if reference-rater training in use of the CAM was explicitly-stated. This follows published recommendations for valid use of the CAM along with diagnostic assignment in delirium research. |
| Information sources* | 7 | Describe all information sources in the search (e.g., databases with dates of coverage and contact with authors to identify additional sources), as well as the date the most recent search was executed. | See appendix |
| Search | 8 | Present the full electronic search strategy for at least 1 database, including any limits used, such that it could be repeated. | See appendix |
| Selection of sources of evidence† | 9 | State the process for selecting sources of evidence (i.e., screening and eligibility) included in the scoping review. | All authors and an academic liaison-librarian were involved in an iterative process to determine search terms. MEDLINE, CINAHL, PsycINFO, EMBASE and SCOPUS databases were searched. Publication date was limited from 1st of January 1996 to 12th of August 2017. A full list of key words and Medical Sub-heading (MeSH) is available see **Appendix.**  Independent title, abstract, full text review and cross check was carried out by MBS and IW, with conflicts resolved by consensus. Where the same study was reported in more than one manuscript, additional information from related or subsequent publications was included where possible. Study heterogeneity precluded meta-analysis but sources of bias and generalisability were assessed using the Quality Assessment of Diagnostic Accuracy Studies (QUADAS) system. Quality considerations and information synthesis was undertaken by all authors.  For the purposes of this study we defined a delirium reference standard as a diagnostic assignment tool such as the World Health Organization (WHO) International Classification of Diseases, 10th Revision (ICD-10) or the American Psychiatric Association Diagnostic and Statistical Manual of Mental Disorders (DSM) criteria, applied by a psychiatrist or consultant physician. The Confusion Assessment Method (CAM) was included as a reference standard for case ascertainment for the purposes of this review, only if reference-rater training in use of the CAM was explicitly-stated. This follows published recommendations for valid use of the CAM along with diagnostic assignment in delirium research. |
| Data charting process‡ | 10 | Describe the methods of charting data from the included sources of evidence (e.g., calibrated forms or forms that have been tested by the team before their use, and whether data charting was done independently or in duplicate) and any processes for obtaining and confirming data from investigators. | Information was charted by hand by authors IW and checked by MBS |
| Data items | 11 | List and define all variables for which data were sought and any assumptions and simplifications made. | See table 1-2 |
| Critical appraisal of individual sources of evidence§ | 12 | If done, provide a rationale for conducting a critical appraisal of included sources of evidence; describe the methods used and how this information was used in any data synthesis (if appropriate). | A critical appraisal using QADAS2 was performed as it was felt this added to the understanding of the existing evidence base and provides useful guidance for improving the evidence base |
| Synthesis of results | 13 | Describe the methods of handling and summarizing the data that were charted. | IW summarized the charted data other authors checked and suggestions were resolved by consensus to achieve clarity and economy |
| **RESULTS** | | | |
| Selection of sources of evidence | 14 | Give numbers of sources of evidence screened, assessed for eligibility, and included in the review, with reasons for exclusions at each stage, ideally using a flow diagram. | See consort diagram |
| Characteristics of sources of evidence | 15 | For each source of evidence, present characteristics for which data were charted and provide the citations. | See table 1 |
| Critical appraisal within sources of evidence | 16 | If done, present data on critical appraisal of included sources of evidence (see item 12). | See table 3 |
| Results of individual sources of evidence | 17 | For each included source of evidence, present the relevant data that were charted that relate to the review questions and objectives. | See tables 1-2 |
| Synthesis of results | 18 | Summarize and/or present the charting results as they relate to the review questions and objectives. | See tables 2 |
| **DISCUSSION** | | | |
| Summary of evidence | 19 | Summarize the main results (including an overview of concepts, themes, and types of evidence available), link to the review questions and objectives, and consider the relevance to key groups. | Of 896 unique studies identified; 91 met full text review criteria. Of 12 eligible studies, four applied recommended case ascertainment to all patients, three used delirium screening tools alone or with case ascertainment tools sub-optimally applied, four used tools not recommended for delirium screening or case ascertainment, one used the Confusion Assessment Method with insufficient information to determine if it met case ascertainment status. Two studies presented delirium incidence rates; 7.8%, and 17% respectively. Prevalence rates ranged from 18-33% for general medical or oncology wards; 42-58% for Acute Palliative Care Units (APCU); and for older cancer patients: 22% and 57%. Three studies reported reversibility; 26% and 49% respectively (APCUs) and 30% (older patients with cancer). Six studies had low risk of bias according to QUADAS criteria; all studies in the APCU setting were rated at higher risk of bias. Tool selection, study flow and recruitment bias reduced study quality. |
| Limitations | 20 | Discuss the limitations of the scoping review process. | Limitations to our review include those related to the methodology of the original studies. Important questions for future work include which tools translate well to inpatient oncology from aged care and stand-alone inpatient palliative care settings, which tools are most suitable for patients, carers and staff, and which reference standards are most appropriate. Establishing a methodical approach to the detection of delirium in clinical and research settings is a prerequisite to determining the incidence, prevalence and reversibility of delirium in this setting. Maintaining a clear accountability for the validation and purpose of the tool, and its use for clinical screening/detection or as a diagnostic reference standard, is essential |
| Conclusions | 21 | Provide a general interpretation of the results with respect to the review questions and objectives, as well as potential implications and/or next steps. | The knowledge base for improved interventions and clinical care in adult oncology is limited by the low number of studies. A clearer distinction between screening and diagnostic tools is required to provide an improved understanding of the rates of delirium and its reversibility in this population. |
| **FUNDING** | | | |
| Funding | 22 | Describe sources of funding for the included sources of evidence, as well as sources of funding for the scoping review. Describe the role of the funders of the scoping review. | No funding was received for this study which was part of M. Philosophy (Medicine) by Dr M B Sands University of Sydney |

JBI = Joanna Briggs Institute; PRISMA-ScR = Preferred Reporting Items for Systematic reviews and Meta-Analyses extension for Scoping Reviews.

* Where *sources of evidence* (see second footnote) are compiled from, such as bibliographic databases, social media platforms, and Web sites.

† A more inclusive/heterogeneous term used to account for the different types of evidence or data sources (e.g., quantitative and/or qualitative research, expert opinion, and policy documents) that may be eligible in a scoping review as opposed to only studies. This is not to be confused with *information sources* (see first footnote).

‡ The frameworks by Arksey and O’Malley (6) and Levac and colleagues (7) and the JBI guidance (4, 5) refer to the process of data extraction in a scoping review as data charting*.*

§ The process of systematically examining research evidence to assess its validity, results, and relevance before using it to inform a decision. This term is used for items 12 and 19 instead of "risk of bias" (which is more applicable to systematic reviews of interventions) to include and acknowledge the various sources of evidence that may be used in a scoping review (e.g., quantitative and/or qualitative research, expert opinion, and policy document).

*From:* Tricco AC, Lillie E, Zarin W, O'Brien KK, Colquhoun H, Levac D, et al. PRISMA Extension for Scoping Reviews (PRISMAScR): Checklist and Explanation. Ann Intern Med. 2018;169:467–473. [doi: 10.7326/M18-0850](http://annals.org/aim/fullarticle/2700389/prisma-extension-scoping-reviews-prisma-scr-checklist-explanation).
