# Supplementary material for: The detection of delirium in admitted oncology patients: a scoping review
Source: Eur Geriatr Med. 2022 Jan 15;13(1):33–51. doi: 10.1007/s41999-021-00586-1 (PMC8860783; doi:10.1007/s41999-021-00586-1)
Supplement: Supplementary file 2 — Supplementary file2 (DOCX 1050 KB) [file 41999_2021_586_MOESM2_ESM.docx]

1. DATABASE SEARCHES
   1. MEDLINE Search August 12^th^ 2017

- 1. SCOPUS Search August 12th 2017

- 1. CINAHL Search August 12^th^ 2017

- 1. PsycINFO Search August 12^th^ 2017
