# Supplementary material for: The detection of delirium in admitted oncology patients: a scoping review
Source: Eur Geriatr Med. 2022 Jan 15;13(1):33–51. doi: 10.1007/s41999-021-00586-1 (PMC8860783; doi:10.1007/s41999-021-00586-1)
Supplement: Supplementary file 3 — Supplementary file3 (DOCX 14 KB) [file 41999_2021_586_MOESM3_ESM.docx]

SQID LIST OF Abbreviations

DELIRIUM ASSESSMENT TOOLS

**4AT** 4 A’s Test

**bCAM** brief Confusion Assessment Method

**CAM** Confusion Assessment Method

**DOSS** Delirium Observational Screening Scale

**MDAS** Memorial Delirium Assessment Scale

**MMSE** Mini-Mental State Examination

**NEECHAM** Neelon and Champagne delirium assessment tool

**Nu-DESC** Nursing Delirium Screening scale

**RADAR** Recognizing Acute Delirium As part of your Routine

**SQiD** Single Question in Delirium

OTHER ABBREVIATIONS

**ADL** Activities of Daily Living

**AKPS** Australian Karnofsky Performance Status

**APACHEII** acute physiology and chronic health evaluation II

**APCU** Acute Palliative Care Unit (i.e. Palliative Care Unit in Acute Hospital or comprehensive cancer centre)

**AUC** Area Under Curve

**CCM** Charlson Co-morbidity (score)

**CI** Confidence Interval

**DSM** Diagnostic and Statistics Manual (editions IV, IVR [revised] and 5)

**ECOG** Eastern Clinical Oncology Group

**ICU** Intensive Care Unit

**IRQ** Inter-Quartile Range

**LOS** Length of Stay (days)

**MLOS** Median Length of Stay (days)

**MeSH** Medical Sub-Headings

**NPV** Negative Predictive Value

**PPV** Positive Predictive Value

**SD** Standard deviation

**WHO** World Health Organisation

**ICD-10** International Classification of Diseases, 10^th^ Revision
